# Occipital-interhemispheric transtentorial pineal mass resection

**DOI:** 10.3171/2023.10.FOCVID23161

**Published:** 2024-01-01

**Authors:** Grant Arzumanov, Seung W. Jeong, Bhavika Gupta, Rocco Dabecco, Jose Sandoval, Matthew J. Shepard, Jody Leonardo, Alexander Yu

**Affiliations:** Department of Neurosurgery, Allegheny General Hospital, Pittsburgh, Pennsylvania

**Keywords:** exoscope, improved ergonomics, pineal tumor, occipital interhemipsheric transtentorial, microscopic excision

## Abstract

The occipital approach for pineal tumors was first described by James Poppen in 1966. Since then, it has been widely used for accessing deep-seated tumors as it offers a wider surgical view than the supracerebellar transtentorial approach. This video demonstrates the technical nuances of the occipital transtentorial approach and the exoscopic dissection of a pineal gland tumor in a 66-year-old male. Use of the exoscope over the microscope provides certain ergonomic advantages and improves surgical workflow, as demonstrated here.

The video can be found here: https://stream.cadmore.media/r10.3171/2023.10.FOCVID23161

## Transcript

This video highlights the benefits of the use of exoscope visualization and difficult patient positioning, in particular, pineal region pathology. Benefits of exoscope visualization include surgeon ergonomics. Surgeon ergonomics are improved once the visualization device is no longer attached to the surgeon. Increased magnification: exoscope devices have the ability for not only optical zoom, but also digital zoom. Due to the independence of the visualization device from the surgeon, a wide range of angles are possible while maintaining surgeon ergonomics. In this example, we demonstrate the ability to visualize and perform adequate microdissection work without changing patient positioning and while maintaining surgeon ergonomics.

### 1:12 Case Presentation.

This is a 66-year-old male who presented with 3 days of altered mental status and confusion. On examination, he was intact besides confused and bilateral upgaze palsy. CT of the head without contrast reveals a hyperdense lesion in the pineal region concerning for a hemorrhagic pineal mass. MRI of the brain with and without contrast demonstrated similar findings. The patient underwent placement of an external ventricular drain for hydrocephalus on arrival. The patient’s confusion improved but the upgaze palsy persisted until after surgery. Cerebrospinal fluid was sent for diagnosis of the pathology but was unremarkable. Given the CSF findings and the lack of a diagnosis, operative intervention was offered.

### 2:04 Risks and Benefits.

Risks of the procedure were discussed with the patient and the family. Risks include, but are not limited to, stroke, hemorrhage, infection, coma, and death. Benefits include the ability to obtain tissue diagnosis, resection of mass, and potentially the treatment of his hydrocephalus from decompression of the cerebral aqueduct. Multiple surgical options were discussed. Endoscopic biopsy and third ventriculostomy were deferred given the likely low diagnostic yield secondary to intratumoral hemorrhage. Third ventriculostomy was also deferred given the anatomy of a high-riding posterior cerebral artery and basilar artery. An infratentorial supercerebellar approach was also considered but deferred given the angle of the tentorium.^1,2^

### 2:55 Patient Positioning.

The patient was positioned in three-point fixation in a three-quarter prone or park-bench position with the right side facing down. An occipital craniotomy with exposure of the superior sagittal sinus was performed. A transtentorial approach was utilized for resection of the pineal mass.^3,4^

### 3:15 Operative Setup.

Here is a bird’s-eye view of the operative setup. Each 3D TV can be placed at the surgeon and surgeon assist’s discretion. The camera arm lies over the shoulder or over the head of the surgeon. The operator can adjust the camera accordingly as well as the 3D TV’s position can be placed in the line of sight of the surgeon. When comparing the traditional microscope and exoscope, surgeon ergonomics are improved as the visualization device is no longer attached to the surgeon. Due to the independence of the visualization device from the surgeon, a wide range of visualization angles are possible, while maintaining the ergonomics of the surgeon. As demonstrated here, the independence of the camera from the surgeon allows the surgeon to remain in relatively the same position while allowing the camera to move according to the pathology at hand. Also, the 3D TVs can be placed according to the surgeon’s preference independent of the camera.

### 4:12 Surgical Approaches to the Pineal Region.

Several factors were taken into account in choice of approach and positioning. These include the angle of the tentorium, location of deep venous drainage in relation to the lesion, neck thickness, and neck mobility. The venous drainage in this case was typical for pineal gland–based pathology. Hence, a supracerebellar approach was ideal for improved avoidance of injury to deep venous structures. The angle of the tentorium on this patient is relatively steep. Considerations of a Concorde position and seated position were considered. Although possible, a Concorde position or seated position may improve the angle of approach but would not eliminate the steep angle of approach in this patient. Of note, we have used exoscopes in the past with these approaches and continue to find them beneficial over traditional microscopes. The transtentorial approach improves the steep angle of exposure but allows the benefits of a supracerebellar approach when considering the avoidance of injury to the deep venous structures. A seated or Concorde approach with the transtentorial approach, as noted here, becomes unnecessary or even becomes more challenging when compared to a park-bench approach, as the approach with the seated or Concorde positioning approaches from an inferior to superior direction, whereas in this case, the approach is more vertically oriented. Neck thickness and mobility is also taken into consideration. Excessive neck flexion of patients with shorter, thicker necks and necks with fusions or decreased mobility from autofusion is not without risk of airway compromise, vascular compromise, and increased venous congestion. Although the transtentorial approach can be somewhat disorienting, orientation is achieved with identification of the transverse sinus, straight sinus, and tentorial edge. Neuronavigation is helpful in these circumstances utilizing MRI and CT venogram. Once the sagittal sinus is identified for the interhemispheric approach, identifying the transverse sinus, straight sinus, and tentorial edge orients the surgeon to the proper dural opening, which in this case is taken lateral to the straight sinus from a posterior to anterior approach to assist in identifying the deep drainage systems.

### 6:30 Surgical Procedure.

Patient is placed in a three-quarter prone position and registered. Patient and surgeon ergonomics are noted. An interhemispheric approach is utilized via drainage of CSF from the cisterns and external ventricular drain. Identification of the straight sinus, transverse sinus, falx, and tentorium was performed. The tentorium was cauterized in a safe location and opened up toward the incisura. The tentorial leaflet is tacked up. Arachnoid is opened over the supracerebellar cistern. Arachnoid planes were maintained identifying the pineal mass. An opening was made in the mass and the mass was debulked.^5^ The ventricular portion of the tumor was identified. The mass was then dissected over the tectal plate. Further mobilization of the tumor was then performed, carefully identifying any venous structures through the dissection. The mass was then amputated, and the final remnants removed. Visualization of the foramen Monro from the third ventricle were made, and also visualization of the aqueduct was performed. Both of these areas were free of clot and debris.

### 9:50 Postoperative Course.

The patient was extubated after surgery with improved confusion and resolved upgaze palsy. At 1-month follow-up, the patient was intact with expected wound healing. Postoperative MRI demonstrated gross-total resection. Postoperative CT venogram demonstrated patent venous sinuses as well as deep drainage system. Final pathology revealed a hemorrhagic pineal cyst.

## Disclosures

The authors report no conflict of interest concerning the materials or methods used in this study or the findings specified in this publication.

## Author Contributions

Primary surgeon: Yu. Assistant surgeon: Arzumanov, Jeong, Sandoval. Editing and drafting the video and abstract: Arzumanov, Jeong, Gupta, Dabecco, Sandoval, Shepard. Critically revising the work: all authors. Reviewed submitted version of the work: Arzumanov, Jeong, Shepard, Leonardo. Approved the final version of the work on behalf of all authors: Yu. Supervision: Yu, Dabecco, Leonardo.

## Supplemental Information

### Patient Informed Consent

The necessary patient informed consent was obtained in this study.
